# Effects of Mental Health Stigma on Clinical Decision-Making in the Context of Digital Medicine

**DOI:** 10.1192/j.eurpsy.2025.1509

**Published:** 2025-08-26

**Authors:** I. Papazova, N. L. Hartmann, J. Grimmer, A. Hasan, N. Khorikian-Ghazari

**Affiliations:** 1 Department of Psychiatry, Psychotherapy and Psychosomatics, Medical Faculty, University of Augsburg, BKH Augsburg; 2 DZPG (German Center for Mental Health), partner site München/Augsburg, Augsburg, Germany

## Abstract

**Introduction:**

People with mental illness often experience stigma and discrimination, which can reduce treatment outcomes and quality of life. Numerous studies have shown that stigmatizing attitudes among physicians negatively affect both psychiatric and somatic care. Recently, technological advancements have led to the emergence of digital medicine as a new avenue for health care. However, little is known about how stigmatizing attitudes toward patients with mental illness might impact clinical decisions in the context of digital medicine

**Objectives:**

This study aims to assess how implicit and explicit stigma against mental illness among medical students and general practitioners affects their decision for recommending treatment through a digital mHealth app.

**Methods:**

A total of 62 general practitioners and 60 medical students participated in the anonymous online survey. After providing demographic information, participants reviewed two case vignettes: one depicting a patient with a comorbid mental and somatic illness, and the other depicting a patient with only a somatic illness. Participants rated, on a scale from 1 to 10, the likelihood of prescribing an mHealth app designed to enhance treatment of the somatic disease. The Social Distance Scale (SDS) and the Implicit Association Test (IAT) were used to measure explicit and implicit stigma, respectively. The IAT is a computer-based task that assesses implicit bias regarding the perceived incompetence associated with psychiatric disorders compared to somatic disorders.

**Results:**

On average, participants were more likely to prescribe an mHealth app for patients with only a somatic illness than for patients with both somatic and comorbid mental illness (p < .001). Furthermore, implicit stigma was a significant predictor of participants’ preference to treat patients with somatic over mental disorders (p = .013). There were no group differences in the IAT score.

**Conclusions:**

Our findings indicate a bias against people with mental illness among both medical students and physicians, even within the context of digital medicine. Future research is needed to further examine the scope and impact of stigmatizing attitudes on patient health care outcome.

**Disclosure of Interest:**

I. Papazova: None Declared, N. Hartmann: None Declared, J. Grimmer: None Declared, A. Hasan Consultant of: Rovi, Recordati, Otsuka, Lundbeck, AbbVie, Teva and Janssen-Cilag, Speakers bureau of: Janssen-Cilag, Otsuka, Recordati, Rovi, Boerhinger-Ingelheim and Lundbeck, N. Khorikian-Ghazari: None Declared

